# Determining the impact of smoking point of sale legislation among youth (Display) study: a protocol for an evaluation of public health policy

**DOI:** 10.1186/1471-2458-14-251

**Published:** 2014-03-14

**Authors:** Sally Haw, Amanda Amos, Douglas Eadie, John Frank, Laura MacDonald, Anne Marie MacKintosh, Andy MacGregor, Martine Miller, Jamie Pearce, Clare Sharp, Martine Stead, Catherine Tisch, Winfried van der Sluijs

**Affiliations:** 1 School of Nursing, Midwifery & Health, University of Stirling, Stirling, UK; 2 UK Centre for Tobacco and Alcohol Studies (UKCTAS), Centre for Population Health, University of Edinburgh, Edinburgh, UK; 3 Institute for Social Marketing, University of Stirling, Stirling, UK; 4 Scottish Collaboration for Public Health Research and Policy, Edinburgh, UK; 5 ScotCen Social Research, Edinburgh, UK; 6 Child & Adolescent Health Research Unit, University of St Andrews, St Andrews, UK; 7 Centre for Research on Environment, Society and Health (CRESH), University of Edinburgh, Edinburgh, UK

**Keywords:** Public health, Evaluation, Legislation, Point of sale advertising, Tobacco, Adolescents, Smoking

## Abstract

**Background:**

Tobacco advertising and product promotions have been largely banned in the UK but point of sale (POS) tobacco advertising is one of the few places where tobacco products may be legitimately advertised. POS displays have been shown to increase susceptibility to smoking, experimentation and initiation into smoking. These displays may also influence perceived prevalence of smoking and the perception that tobacco products are easily obtained and are a ‘normal’ product. A ban of POS tobacco advertising was introduced in Scotland in large tobacco retail outlets of over 280m^2^ internal sales floor areas (mainly supermarkets) in April 2013 and will be extended to include smaller tobacco retail outlets in April 2015. However, the impact of POS bans on smoking attitudes, behaviours and prevalence has yet to be determined.

**Methods/design:**

This study has a multi-modal before and after design and uses mixed methods to collect data, at baseline and then with longitudinal follow-up for 4 years, in four purposively selected communities. For the purposes of the study, community is defined as the catchment areas of the secondary schools selected for study. There are four main components to the on-going study. In each of the four communities, at baseline and in follow-up years, there will be: mapping and spatial analyses of tobacco retail outlets; tobacco advertising and marketing audits of tobacco retail outlets most used by young people; cross-sectional school surveys of secondary school pupils; and focus group interviews with purposive samples of secondary school pupils. The tobacco audit is supplemented by interviews and observations conducted with a panel of tobacco retailers recruited from four matched communities.

**Discussion:**

This study examines the impact of the implementation of both a partial and comprehensive ban on point of sale (POS) tobacco advertising on attitudes to smoking, brand awareness, perceived ease of access to tobacco products and youth smoking prevalence. The results will be of considerable interest to policy makers both from the UK and other jurisdictions where they are considering the development and implementation of similar legislation.

## Background

Tobacco advertising and marketing activity have been shown to have a direct impact on adolescent smoking intentions, perceived smoking prevalence and youth smoking prevalence [1,2]. In addition, a dose–response relationship has been demonstrated between adolescent tobacco marketing awareness and smoking uptake [3]. Following an EU directive [4], the Tobacco Advertising and Promotion Act (TAPA) was implemented in the UK between 2003 and 2005 [5]. The legislation bans advertising on billboards, in cinemas and print media, by direct mail, on-pack promotions, and through brand sharing and international tobacco sponsorship. On 29^th^ April 2013, point of sale (POS) tobacco advertising was banned in large retailers in Scotland [6]. POS advertising in smaller retail outlets is now one of the few ways in which the tobacco industry can legitimately promote their products in Scotland. The ban will be extended to include these retail outlets in April 2015 [6]. The gantries currently used to display tobacco products are usually supplied by the tobacco industry and sited in prominent in-store positions, most often at checkouts, with products arranged attractively and sometimes positioned in such a way as to obscure health warnings [7]. Recent UK research [8,9] and a systematic review [10] have found that, in children, POS displays increase susceptibility to smoking, experimentation and initiation into smoking. These displays may also influence perceived prevalence of smoking and the perception that tobacco products are easily obtained and are a ‘normal’ product. Studies of adults suggest that POS advertising increases impulse cigarette purchases [11]. Positioning of POS displays may also be important in that cigarette retailers located in communities with a high proportion of children have been shown to be more likely to display cigarettes near children’s products [12]. A multi-centre Canadian study also found that stores near schools with high smoking prevalence had significantly lower mean price per cigarette, more in-store promotions (price, gift or bonus promotions) and fewer government-sponsored health warnings [13].

There are a growing number of jurisdictions, such as Ireland, Iceland, Thailand, and some provinces and territories in Canada [14], where POS bans have been introduced but few studies of the impact of POS bans have been conducted. An exception is a study of the Irish legislation [14], which found that high levels of compliance (97%) were accompanied by increasing support for the law, a reduction in recall of displays among both adults (49% to 22%; p < 0.001) and young people (81% to 22%; p < 0.001), a reduction in perceived youth smoking prevalence among young people, and an increase in beliefs that the law made it easier for adults to quit smoking or for children not to initiate smoking. The study failed to find a reduction in smoking prevalence either among young people or adults. However, the short follow-up period (one month) and the small sample sizes, particularly for young people (n = 214), made it unlikely that a reduction in smoking prevalence would be detected and prevents any conclusions about the longer-term impacts of the Irish legislation being drawn.

### Study aims

In this study we assess the impact of Scottish legislation to ban point of sale (POS) tobacco advertising on young people’s exposure to tobacco advertising, their attitudes towards smoking and their smoking behaviour. We will also identify any ‘unintended consequences’ associated with the implementation of the legislation.

### Intervention

The intervention to be evaluated is the prohibition of tobacco advertising at point of sale (POS) contained in Sections 1 to 3 of the Tobacco and Primary Medical Services (Scotland) Act 2010 (TPMS Act) [6]. The legislation prohibits the display of tobacco products or tobacco-related products in places where tobacco products are offered for sale and requires retailers to conceal cigarettes from general view, either by covering up cigarette gantries/dispensers or by storing cigarettes under the counter. Under the legislation, displays of tobacco products or tobacco-related products (Section 2) and prices (Section 3) are also considered to be advertisements. The overall policy objective of the legislation is to reduce the attractiveness of tobacco products among children and adolescents under the age of 18, which in turn may lead to a reduction in initiation into smoking and in the longer term a reduction in smoking prevalence. The legislation was implemented in large retail outlets (mainly supermarkets) with over 280m^2^ of internal floor area used for the display of goods and serving of customers on 29^th^ April 2013 and will be extended to the remaining smaller tobacco retail outlets in April 2015.

### Research questions

Our specific research questions are:

1. Does implementation of POS regulations in the TPMS Act in i) supermarkets alone (partial ban); and ii) all tobacco retailers (complete ban) result in changes in exposure to tobacco advertising in young people aged 12 to 17 years?

2. Is a reduction in exposure to POS advertising associated with: changes in brand awareness; perceived accessibility of tobacco; perceived prevalence of youth smoking; susceptibility to smoking; and the incidence and prevalence of smoking in young people aged 12 to 17 years?

3. Is there any evidence of socio-economic patterning in any of the attitudinal or behavioural outcomes in young people?

4. What is the association between area-level deprivation and i) levels of POS tobacco advertising and availability of cigarettes pre-and post-legislation; or ii) enforcement of the legislation when implemented?

5. Is there any evidence of a dose-response relationship between changes in exposure to POS advertising and interim and longer-term outcome measures in young people?

6. Are there any unintended adverse consequences associated with the legislation, for example, an increase in cigarette purchases from black-market sources?

7. Is there any evidence of changes in POS advertising and marketing strategies in the lead up to implementation of measures in the TPMS Act in either supermarkets or small retailers?

## Methods/design

The study has a multi-modal before and after design and uses mixed methods to collect data in four purposively selected communities. For the purposes of the study, community is defined as the catchment areas of the secondary schools selected for study. Schools were purposively selected to reflect two levels of urbanisation (large urban vs. small town) and two levels of socio-economic deprivation (high vs. medium or low). Deprivation categories were derived from the population-weighted mean Scottish Index of Multiple Deprivation (SIMD) score for all data zones falling within the school catchment areas and the proportion of children from each school receiving free school meals. To keep the influence of school factors, other than urbanisation and deprivation, to a minimum, the selected schools were non-denominational local authority schools on mainland Scotland with an ethnic minority population of less than 10% of the school roll. In addition, schools were also selected to have a pupil roll of between 1100 and 1200.

There are four main components to the study. In each community we are conducting:

•Mapping and spatial analysis of the location and density of tobacco retail outlets.

•Tobacco advertising and marketing audits of tobacco retail outlets most used by young people, supplemented by interviews and observations with a panel of retailers in four matched communities.

•Cross-sectional school surveys of pupils, with five embedded pupil cohorts.

•Focus group interviews with purposive samples of pupils.

Data for the study components were collected at baseline between February and April 2013, prior to implementation of the legislation on 29^th^ April 2013. Follow-up data collection will be repeated annually for four years. Additional marketing audits were conducted immediately post-legislation in May 2013 to assess compliance in large retail outlets and this will be repeated in May 2015 in smaller retail outlets, following implementation of the POS legislation in smaller retailers in April 2015.

Details of the study components are as follows:

1. Annual mapping and spatial analysis studies of tobacco retail outlets

Data (including address and full postcode) for all tobacco retailers in the study communities were extracted from the tobacco retailers register (http://www.tobaccoregisterscotland.org) and mapped at baseline (January 2013) and then verified through field visits during which every street in the four communities was inspected [15].

Baseline tobacco outlet data were geo-coded (using Code-Point®) to provide geographical coordinates and then integrated into a Geographical Information System (GIS). These data will be combined with data from the marketing audits (see below) and analysed to provide an assessment of changes in tobacco retailing and advertising over the study period. In the analysis of POS exposure, we will limit our focus to supermarkets, off-licences and retailers most likely to sell cigarettes to young people, including confectioners, tobacconists and newsagents, grocers (including licensed), petrol stations, and fish and chip shops.

The data from the mapping and spatial analysis studies will be used to:

•Monitor the number and rate (per population) of tobacco outlets in each of the four communities at baseline and in follow-up years.

•Examine whether there is geographical clustering of tobacco outlets around secondary schools and whether there are changes in clustering over the study period. Using methods trialed by one of the research team (JP) in previous work [16], we will examine the spatial clustering of tobacco outlets within 1.5 km of each school.

•Develop tobacco retailing and advertising exposure measures for each secondary school pupil participating in the study based on a weighted average of tobacco outlets in the buffer surrounding their school and home environments and exposure index scores calculated for each retail outlet using baseline audit data. Mean density estimates will be calculated for 1.5 km buffers around the school and home environment of each secondary school participant.

•Calculate changes in exposure to tobacco products and advertising for the full sample and for each of the four communities individually, stratified by neighbourhood deprivation and urban rural status.

•Provide a verified list of outlets to be visited and observed as part of the discreet audit of all retailers in each community (see below). Annual auditing of retail outlets (see below) will maintain the accuracy of the number and rate of outlets selling tobacco in each community over the study period.

2. Annual tobacco advertising and marketing audits

As with the mapping studies, the tobacco advertising and marketing audits focus on supermarkets, off-licences, confectioners, tobacconists and newsagents; grocers (including licensed), petrol stations, and fish & chip shops. There are two parts to this study component:

i) Retailer Panel: A panel of 24 retailers (representing the main retail types) has been recruited from communities matched to our four main study areas in order to monitor POS displays and related marketing activity. We chose not to recruit the retailer panel from the main study area, in order to minimise the likelihood that the identity of the study areas was made public and thereby compromising the study integrity. Similarly, large supermarkets were excluded from the panel sample for the same reason. Each outlet was visited, at baseline (Feb-Apr 2013), to collect observational data on POS advertising and marketing strategies using an adapted version of the form developed to monitor the impact of the Tobacco Advertising and Promotion Act (TAPA) [16].

In addition to the in-store observations, in-depth interviews have also been conducted with retail managers/owners from each outlet. These were audio recorded and will be repeated annually to explore their views and experiences before, during and after the implementation of the POS ban, to assess changes from the retailer’s perspective, explore their experiences as they prepare to and eventually implement the POS ban, and identify any problems that arise and how retailers deal with these. Additionally, the data will enable us to understand how the nature of the sales process changes and examine how customers deal with a new procedure for asking for cigarettes. The interviews will also be used to explore under-age sales and the perceived impact of the legislation on proxy sales.

ii) Discreet Audit: The discreet audit includes all tobacco retail outlets in the study communities that fall into the six categories identified above. Baseline data (February to March 2013) were collected by experienced observers, who visited all outlets in pairs to record brief information on tobacco product availability and display. The brief information included data on the visibility and placement of tobacco products within the store; whether and how tobacco products are displayed; whether and how tobacco products are actively promoted for sale (both external and internal); branding of display units and pack sizes available; most prominent brand, if any; communication and visibility of pricing information; and tobacco control signage. The audits did not require retailer co-operation and observers devised techniques to accurately recall and unobtrusively record marketing and advertising information.

The discreet audit will be repeated annually until 2017. Additional visits were made to supermarkets as part of the discreet audit in May 2013 to assess immediate compliance with the legislation. In May 2015, similar visits will be made to all small shops affected in by the legislation, in order to assess compliance amongst smaller retailers. Data collected will be used to develop a metric for POS exposure with measures developed to assess location, size, proximity and visibility of displays from key reference points such as till-points, and entrance areas. Where appropriate, measures will be developed with the aid of visual prompts, for example to indicate the relative visibility of the display. It is anticipated that POS exposure will be affected by a number of factors including increased industry activity, particularly in the lead-up to full implementation in 2015, and by retailer non-compliance (e.g. delays in removing gantries or gantries being reused for other non-tobacco products) or poor implementation (e.g. leaving sales shutters open after a sale is made). As well as assessing exposure, the audit will also assess level of compliance with both the current and new point of sale legislation, along with any evidence of strategies used to circumvent the legislation.

Data governance requires that the identity of all retail sites audited and all panel participants approached to take part in an interview remain confidential. In line with these requirements all sites and participants have been assigned non-identifiable codes to retain anonymity. Identifiable data (e.g. participants, premise names and address details) are held on a separate database and will be linked to electronic data files using these non-identifiable codes. Technical reports, presentations and publications will ensure that no participants or retail premises can be identified.

3. Annual school surveys of secondary school pupils

The school survey has a repeat cross-sectional design with embedded cohorts (Additional file 1: Table S1). We hypothesise that implementation of a partial ban on POS adverting (larger retailers only) will have only a small impact on awareness and attitudes. In order to assess this assertion, baseline surveys of S2 (age 13) and S4 (age 15) pupils were conducted in each of the four study schools between February and March 2013. This survey will be repeated one year later. The impact of the Scottish partial POS ban on 13-and 15-year-olds will then be compared with the impact on a similar age group of the comprehensive POS ban in Ireland, where legislation was implemented simultaneously in both large and smaller retailers in 2011 [14].

To assess the impact on the implementation of POS legislation in smaller retailers in 2015, a second baseline survey will be conducted with all pupils (S1-6) from our study schools in February to March 2015, with repeat surveys conducted annually for two years post-implementation. This survey series will allow us to measure the impact of a comprehensive ban (supermarkets and smaller retailers) on POS advertising on behavioural outcomes including smoking incidence and prevalence, as well as on brand awareness and other attitudinal outcomes.

In all survey waves, after ‘opt-out’ consent has been provided by parents and pupils, data will be collected using an anonymous self-complete questionnaire administered by class teachers under exam conditions. The questionnaire contains questions on personal smoking behaviours and attitudes towards tobacco use as well as family and peers’ behaviours and attitudes, access to tobacco products, brand awareness and exposure to tobacco advertising. An additional School Level Questionnaire (SLQ) intended for the head teacher or deputy head teacher will be used to gather information on the characteristics, resources and health-promoting aspects of all the participating schools.

4. Annual focus group interviews with purposive samples of S2 and S4 school pupils.

At baseline in March 2013, 16 focus groups were conducted with S2 and S4 pupils in each study community. They were all single-sex groups, with between 3 and 9 participants and lasted between 30–50 minutes. Table 1 gives the sample structure. The focus groups were conducted one and two weeks after the school survey (so that the pupils’ discussions did not influence questionnaire responses) and audio-recorded with the permission of group participants. The topic guide included: general discussion about the community; leisure time activities; local smoking behaviours and cultures; access to tobacco products including direct, indirect/proxy and black-market; awareness of and views on tobacco promotion including point of sale, other direct marketing methods, packaging, branding; awareness and perceptions of the impact of the legislation; and views about preventing youth smoking.

**Table 1:** Focus group sample structure

	**High deprivation**		**Medium/low deprivation**		**Total**
	S2	S4	S2	S4	
Urban	2	2	2	2	8
Semi-urban	2	2	2	2	8

Focus group participants were recruited with the help of teachers in the study schools, to include young people who are smokers or have regular contact with smoking, such as having smoking friends or living in a home with smoker(s). The aim was to include young people who are most at risk of becoming adult smokers. These recruitment methods have been used successfully in a recent study by one of the research team (AA) on young people’s sources of cigarettes [17]. We used ‘opt-out’ consent for pupils identified as potential focus group participants separate from but using a similar strategy to that used in the school survey phase.

The focus groups will be repeated annually until 2017 and will provide more detailed and nuanced contextual information and insights into young people’s experiences and perceptions.

### Outcome measures

**Figure 1:**
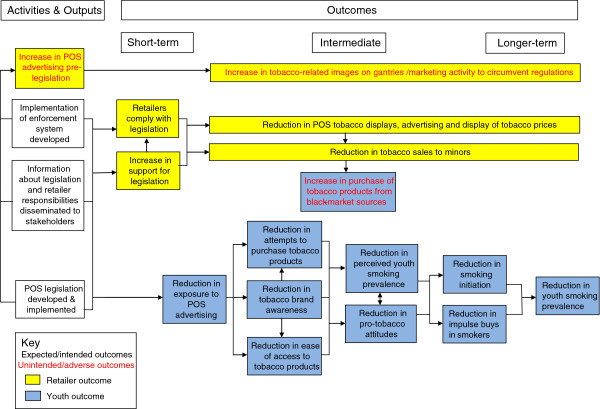
Logic Model of Activities, Outputs and Outcomes Associated with Point of Sale (POS) Legislation.

A logic model provides the framework for the evaluation (see Figure 1). This proposes causal pathways that link together the implementation of the POS legislation with a set of short-term, intermediate and long-term outcomes which will be assessed by the various study components. Therefore, rather than defining primary and secondary outcomes, we have set out a timeframe within which we believe the outcomes will occur. We have classified outcomes as *short-term*, if they were likely to occur within 3 months of implementation of the legislation*; intermediate*, if they were likely to occur up to one year post-implementation; and *longer-term* if they were likely to occur more than a year post-implementation.

Short-term outcomes of interest are prevalence of POS advertising in tobacco retail outlets (assessed by components 1 and 2) and exposure to POS advertising (assessed by components 1, 2 and 3).

Intermediate outcomes of most interest are awareness of point of sale advertising, cigarette brand awareness, perceived ease of access to cigarettes, attempts to purchase cigarettes, perceived youth smoking prevalence, and pro-tobacco attitudes. Long-term outcomes of most interest are: incidence and prevalence of smoking.

Data obtained through interviews with members of the retailer panel (component 2) and focus group discussions with pupils will provide qualitative data for all the intermediate outcomes.

In addition to the outcomes outlined above, the study design will also enable us to identify any unintended or adverse consequences associated with the legislation, such as an increase in purchases from black market sources or proxy purchases; and the introduction of new strategies by retailers or the tobacco industry to circumvent the regulations. In Ireland, for example, images of tobacco-related paraphernalia such as cigarette lighters regularly appear on the blank covers of the cigarette gantries.

### Sample size and power calculations for school surveys

Table 2 below gives our estimated sample sizes and smoking prevalence and incidence for each of the school survey waves. The numbers of current and ever smokers are extrapolated from data from the 2008 Scottish Schools Adolescent Lifestyle and Substance Use Survey (SALSUS) National Report [18], the Health Behaviours of School Children (HBSC) 2010 Scotland National Report [19], and the 2008 Scottish Health Survey Data [20].

**Table 2:** Estimated sample sizes and smoking prevalence and incidence by age and year group

**Age group/Grade**	**% pupils in each class**^**1**^	**N pupils per school**	**Tot no pupils 4 schools**	**Estimated response rates**^**2**^		**Prevalence regular smoking**^**3**^		**Incidence regular smoking**	**Prevalence ever smoked**		**Incidence ever smoked**
				**%**	**N**	**%**	**N**		**%**	**N**	
School Surveys 2013-14											
13/S2	100%	188	752	85%	640						
15/S4	100%	188	752	85%	640						
Total all schools		376	1504	85%	1280						
Total per school			376	85%	320						
School Surveys 2015-17											
12/S1	18.1%	217	866	85%	736	1.0%	7	1.0%	4%	26	4%
13/S2	18.3%	220	878	85%	747	4.8%	35	3.8%	23%	168	19%
14/S3	18.8%	225	902	85%	767	11.0%	84	6.3%	35%	268	13%
15/S4	18.8%	225	901	85%	766	16.5%	126	5.5%	44%	333	9%
16/S5	15.7%	189	754	85%	641	20.0%	128	3.5%	47%	298	3%
17/S6	10.2%	123	491	85%	417	24.0%	100	4.0%	47%	194	0%
Total all schools		1198	4793		4074		482			1287	
Per School			1198		1018		120			322	

1. Distribution of pupils over different age groups is based on most recent School statistics released by Scottish Government.

2. Response rates are based on HBSC survey experience.

3. Regular smoking defined as weekly smoking. Percentage 13 and 15-year old based on SALSUS, Percentages 12, 14, 16 and 17-year olds based on plotting, making use of HBSC smoking data for 11-year olds, SALSUS 13 and 15-year olds and 17-year olds’ data from the Scottish health survey (16-24-year olds: Male 24%; Female 29%) [18-20].

Table 3 provides detailed power calculations for cross sectional analyses of the main intermediate and long term outcomes for i) the surveys of S2 and S4 school children that will be conducted in 2013 and 2014 and, ii) the whole school surveys that will be conducted between 2015 and 2017. The estimates are based on an average school roll of 1200 pupils and 85% attendance on the day of the survey.

**Table 3:** Power calculations for intermediate and long-term outcomes

			**Sample size required at 0.80 power with two-tailed test**		**Sample before and 1 year after POS Supermarket (4 S2 & 4 S4 from 4 schools 24 pupils each class 85% response rate)**		**Sample before Small Shops POS and 2 years after (all pupils attending schools, 4 schools, school size 1200 pupils, response rate 85%)**	
	**Outcome**	**Change**	**p < .05**	**p < .01**	**4 schools combined**	**Urbanisation (2 levels)**	**4 schools combined**	**Urbanisation (2 levels)**
						**Deprivation (2 levels)**		**Deprivation (2 levels)**
						**Age (2 levels)**		
**All (community population)**					**N = 1280**	**N = 640**	**N = 4074**	**N = 2037**
	Access to tobacco: If try buy, likely to be successful^1^	32%-25%	680	999	>0.80	0.80	>0.99	>0.99
	Access to tobacco: If try buy, likely to be successful^2^	32%-22%	328	479	>0.99	>0.80	>0.99	>0.99
	Awareness tobacco marketing in shops^1^	81%-22%	14	19	>0.99	>0.99	>0.99	>0.99
	Awareness tobacco marketing in shops^2^	81%-71%	305	445	>0.99	>0.99	>0.99	>0.99
	Perceived prevalence regular smoking^1^	62%-46%	164	238	>0.99	>0.99	>0.99	> 0.99
	Perceived prevalence regular smoking^2^	62%-52%	404	591	>0.99	>0.80	>0.99	>0.99
	Incidence regular smoking^3^	4%-2%	1239	1797	>0.80		>0.99	>0.80
	Incidence any smoking^3^	9%-7%	2987	4396			>0.80*	
	Prevalence regular smoking^3^	13%-10%	1841	2707			>0.99	>0.80
	Prevalence regular smoking^3^	13%-9%	1009	1478	>0.80		>0.99	>0.80
**Current smokers**					**N = 136**	**N = 68**	**N = 482**	**N = 241**
	Purchase tobacco from shops^4^	55%-43%	412	603			>0.80	0.82*
	Purchase from Supermarkets^4^	12%-5%	389	563			>0.80	0.85*
	Purchase from Small Shops^4^ (increase after POS Supermarkets	44%-56%	411	603			>0.80	0.82*
	Purchase from Small Shops^4^	44%-32%	393	575			>0.80	0.84*
	Access to tobacco: If try buy, likely to be successful	32%-20%	408	596			>0.80	0.83
	Awareness tobacco marketing in shops^1^	81%-22%	14	19	>0.99	>0.99	>0.99	0.99
	Awareness tobacco marketing in shops^2^	81%-71%	305	445			>0.80	0.80*
	Perceived prevalence regular smoking^2^	62%-50%	404	591			0	0.83*
**Ever smokers**					**N = 422**	**N = 212**	**N = 1287**	**N = 644**
	Purchase tobacco from shops^4^	23%-16%	530	776	>0.80*		>0.99	0.87
	Purchase from Supermarkets^4^	4%-1%	489	697	>0.80*		>0.99	0.85*
	Purchase from Small Shops^4^ (increase after POS Supermarkets )	18%-25%	568	832	>0.80*		>0.99	0.85
	Purchase from Small Shops^4^	18%-12%	588	859	>0.80*		>0.80	>0.80*
	Access to tobacco: If try buy, likely to be successful^2^	32%-22%	408	596	>0.80		>0.99	>0.80
	Awareness tobacco marketing in shops^1^	81%-22%	14	19	>0.99	>0.99	>0.99	>0.99
	Awareness tobacco marketing in shops^2^	81%-71%	305	445	>0.80		>0.99	>0.99
	Perceived prevalence regular smoking^2^	62%-52%	404	591	>0.80		>0.99	>0.80

1 Change based on findings McNeil et al. [14] paper on the removal of Point of Sale tobacco displays in Ireland.

2 Starting percentage based on findings McNeil et al. [14] paper on the removal of Point of Sale tobacco displays in Ireland.

3 Starting percentages based on findings latest published results from HBSC [19] and SALSUS [18] studies.

4 Starting percentages based on findings latest published results from SALSUS [18].

* Denotes power calculation for a one-tailed test. Blank cells are underpowered to detect a significant change.

The estimated baselines and changes in access to tobacco; awareness of tobacco marketing; perceived prevalence of youth smoking; and ease of access to tobacco are based on the evaluation of POS ban in Ireland. [14] The baseline percentages for other intermediate outcomes outlined in below are derived from data from the 2008 Scottish Schools Adolescent Lifestyle and Substance Use Survey (SALSUS) National Report [18] and the Health Behaviours of School Children (HBSC) 2010 Scotland National Report [19]. Power calculations are given for all pupils (community populations), current smokers and ever smokers. An asterisk denotes power calculation for a one-tailed test. Blank cells are underpowered to detect a significant change.

### Data analysis

An advantage of adopting a spatial approach to data collection is that the various quantitative datasets can be readily integrated into a GIS. By collecting postcodes, the retail outlets, audits (retailer panel and discreet) and the school survey information will each be geographically referenced. This will enable us to integrate the data into a single database for further quantitative analysis.

The primary analyses around implementation in large retailers will focus on estimating the reduction in POS advertising between February and April 2013 (baseline) and February-to April 2014 and the relationship between POS exposure and awareness of POS advertising and changes in perceived access to tobacco and perceived youth smoking prevalence. In addition to the above, the primary analyses around implementation in smaller retailers will also examine the impact of full implementation on behavioural outcomes including purchase of tobacco products and smoking incidence and smoking prevalence. Specifically, we will:

•Examine changes in exposure to tobacco advertising, access to tobacco products and attitudes towards smoking between baseline (February to April 2015) and the same months in 2016 and 2017 for the total sample, with sub-group analyses by community deprivation, urbanisation and baseline availability of cigarettes.

•Examine changes in incidence of regular smoking and smoking prevalence between baseline (February to April 2015) and the same months in 2016 and 2017, with sub-group analyses by community deprivation, urbanisation and baseline availability of cigarettes.

•Assess if there is a of a dose-response relationship between changes in POS advertising or changes in availability of cigarettes and other study outcomes. To do this, we will create continuous dummy variables for various measures of advertising exposure which can be used in the analyses.

Given the number of outcomes we wish to examine, in the analyses we will set a higher statistical significance threshold (type I or α error) where possible.

Focus group interviews will be fully transcribed and the data entered into the qualitative computer package NVivo, version 10. The data will be coded and will undergo inductive thematic analysis employing constant comparison to identify key themes, focussing on uncovering the social worlds of the participants, and examples of differing views and experiences. The findings will be used to interpret findings from the quantitative components, in particular, any differences associated with community deprivation or baseline availability of cigarettes, or baseline availability of cigarettes through retail and/or black-market sources. Findings from the focus groups will be reviewed annually in order to identify emergent issues that should be explored further in the school survey.

After each wave of data collection, quantitative and qualitative data will be synthesised using a multi-level approach. First, quantitative data from the mapping, retailer audit and school surveys will be synthesised (Synthesis 1) followed by a synthesis of qualitative data from the focus groups and additional qualitative data from other study components (Synthesis 2). The products of syntheses 1 and 2 will then be combined using a series of mixed methods matrices, which allow the juxtaposition of findings from the different components of the study. We will then use these to generate a narrative synthesis (Synthesis 3). The focus in Synthesis 3 will be on consistencies and contrasts in the data which will form the basis for short interim reports. Once data collection is complete, the synthesis of data across all the study waves will follow a similar process but the focus of the analyses will change over time.

Findings from our study will be placed in a broader context through comparison with national level data. In particular, we will compare levels of compliance in tobacco retail outlets in our study communities with national data collected by Trading Standards Officers. Using data from the national register of tobacco outlets, we will also compare the density (and changes in density) of outlets in the four study communities to all other communities across the country. In addition, findings about changes in availability and sources of cigarettes for under-age smokers for our study populations will be compared with national data available from the Scottish Adolescent Lifestyle and Substance Use Survey (SALSUS) [18].

## Discussion

The POS legislation presents a unique opportunity to examine the impact of a public health policy and, as far as we are aware, this is the first study to robustly assess the impact of a partial and comprehensive ban of POS tobacco advertising on youth attitudes to smoking, perceived access to tobacco products and smoking prevalence. In addition, we will also monitor compliance with the legislation and changes in tobacco retailing and marketing activity, as well as any unintended consequences associated with the legislation, such as new tobacco industry tactics and black-market sales. We have developed a systematic and robust evaluation that overcomes many of the methodological problems inherent in a natural experiment. Through careful design of a multi-component study we will be able to assess differential impacts associated with the legislation by both community deprivation and individual measures of socio-economic status. We will also develop novel approaches, both to the measurement of exposure to point of sale tobacco advertising and depicting changes in spatial and temporal distribution of some of the potential determinants of health inequalities.

The major methodological challenge associated with evaluating the impact of the POS legislation is that it came into force simultaneously across all regions in Scotland. Therefore, neither randomisation nor the use of geographical controls is possible. Instead, we chose an uncontrolled before and after design as the only feasible design. This poses a threat to internal validity and the inference of causation, however, a number of measures to overcome this problem have been included in the design. We have included a set of outcomes on other health behaviours in the school survey, which are unrelated to the legislation and assess change over time in our community populations, thus providing an internal control. We will also assess whether there is a dose response relationship between our measures of tobacco marketing exposure and our short-, intermediate and long-term outcomes - a powerful indicator of cause and effect [21]. In our interpretation and synthesis of the data we will use triangulation, placing greatest weight on outcomes which are confirmed by multiple data sources [22]. Finally, we will collect information on changes in policy/practice and other confounding factors both locally and nationally that could influence the outcomes. This will allow us to eliminate alternative interpretations of the study findings before inferring causation.

Another large-scale evaluation of public policy, the evaluation of Scotland’s smoke-free legislation [23], had a significant impact on the implementation of smoke-free legislation in other jurisdictions. Should the evaluation find that POS legislation has an impact on brand awareness in young people, initiation into smoking and/or smoking prevalence, then this will encourage policy makers in other jurisdictions to develop and implement similar legislation.

### Ethics and data governance

An ethics review determined study integrity to be dependent upon retaining the anonymity of study participants and areas, including matched areas. Necessary measures concern non-disclosure and protection of all local identifiers; local organisations (including schools, retail outlets, education authorities and councils); named individuals associated with these and other local organisations (including school staff, pupils and parents, local authority workers and council representatives, and local retail owners and employees); and the address and contact details associated with these and other local organisations (including postcodes, street names, email addresses, websites and telephone numbers). The protected period relates to the period over which study data are collected to March 2017, and the period necessary for completion of the analysis and publication to December 2019.

### Ethical approval

Ethical approval for the study and its components was obtained from the University of Stirling Management School Ethics Committee; Edinburgh University School of Geoscience Research Ethics Committee; NatCen Research Ethics Committee; and St Andrews University Teaching and Research Ethics Committee.

## Abbreviations

GIS: Geographical information system; POS: Point of sale; SIMD: Scottish index of multiple deprivation; TAPA: Tobacco advertising and promotions act; TPMS (Scotland) Act: Tobacco and Primary Medical Services (Scotland) Act.

## Competing interests

The authors declare they have no competing interests.

## Authors’ contributions

SH, AA, DE, JF, AMM, AM, JP, MS, WvdS designed the study and contributed to the drafting of the manuscript. LM, MM, CS, CT contributed to the drafting of the manuscript. All authors read and approved the final manuscript.

## Pre-publication history

The pre-publication history for this paper can be accessed here:

http://www.biomedcentral.com/1471-2458/14/251/prepub

## Supplementary Material

Additional file 1: Table S1 Repeat cross-sectional school surveys with embedded cohorts. Click here for file
