# Supplementary material for: Determining the impact of smoking point of sale legislation among youth (Display) study: a protocol for an evaluation of public health policy
Source: BMC Public Health. 2014 Mar 14;14:251. doi: 10.1186/1471-2458-14-251 (PMC4004271; doi:10.1186/1471-2458-14-251)
Supplement: Additional file 1: Table S1 — Repeat cross-sectional school surveys with embedded cohorts. [file 1471-2458-14-251-S1.pdf]

Table 1 Repeat cross-sectional school surveys with embedded cohorts

Year of Survey	S1	S2	S3	S4	S5	S6	Phase of Legislation
2013							Baseline pre-implementation in large supermarkets
2014							Post-implementation in large supermarkets (partial ban POS)
2015							Post-implementation in large supermarkets (partial ban POS)<br>Pre-implementation smaller retailers
2016							Post-implementation in smaller retailers (comprehensive ban POS)
2017							Post-implementation in smaller retailers (comprehensive ban POS)
